# Has the *Striga* problem been solved? A field perspective critique of recent progress

**DOI:** 10.1002/ps.70284

**Published:** 2025-10-23

**Authors:** Jonathan Gressel

**Affiliations:** ^1^ Plant and Environmental Sciences Weizmann Institute of Science Rehovot Israel

**Keywords:** S*triga*, biocontrol, suicidal germination, strigolactones, resistance breeding, gene editing

## Abstract

Three root‐parasitic witchweed (*Striga*) species can cause up to total loss of grain and legume crops for millions of farmers in Africa. The damage is initiated before *Striga* emerges from the soil. Four decades of resistance breeding, especially maize, have at best conferred localized moderate resistance that has not greatly increased yields. Recent innovative basic research has led to major advances that are herein evaluated in biocontrol, formulating suicidal germination stimulants and gene‐edited crops. A mycoherbicide was able to become a commercial success because the innovators rendered the fungus hypervirulent, inexpensively cultivated it, formulated it as a seedcoat with ample nutrition for extensive growth, and determined target species specificity for regulators. A slow‐release formulation of suicidal germination stimulants led to a *Striga‐*free crop, but crop planting had to be impractically delayed by almost 3 months for the technology to work. Elegant basic research led to gene‐edited crop plants secreting vastly reduced levels of *Striga* germination stimulant, leading to *Striga asiatica‐*free fields under low seeding levels of the parasite. It is doubted that the results will carry over to fields infested with the far more virulent *Striga hermonthica* having seedbanks so large that the residual amount of stimulant emitted may result in heavy infestation. The editing approach should be extended to suppressing the genes responsible for *Striga* attachment and establishment, and the genes stacked for more robust resistance. Solving only the *Striga* problem is insufficient because farmers with *Striga* are also confronted with insect, pathogen and climate constraints that need simultaneous solutions. © 2025 The Author(s). *Pest Management Science* published by John Wiley & Sons Ltd on behalf of Society of Chemical Industry.

## Graphical abstract

The potential field efficacy of recently proposed breeding, biocontrol, suicidal germination, gene editing and transgenic solutions for *Striga* control is critically evaluated and mostly found wanting improvements.

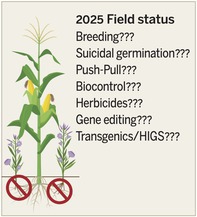

## 1 INTRODUCTION

Root parasitic Orobancaceae are pernicious weed problems throughout the subtropical regions of Africa, Europe and Asia. Of these, three *Striga* spp. (witchweeds) can cause up to total loss of grain and legume crops for millions of farmers, especially in the subsistence agricultural areas of sub‐Saharan Africa. *Striga* infestations are a major reason why grain yields in Africa are a fraction of world averages. Interestingly, hemi‐parasitic *Striga* spp., which perform photosynthesis after they emerge from the soil, are more damaging to crops weight for weight than the related holoparasitic *Orobanche/Philipanche* species, which are wholly dependent on the crop for their organic nutrition. This is probably due to the poisoning (bewitching) of the crop shoots and leaves, providing more sunlight after *Striga* emerges from below ground and becomes photosynthetic.

This review was intended to critically evaluate whether the results of recent basic research are applicable in their present form for use in the field, and if not, what needs to be done to attain effective *Striga* management. For brevity, this review was limited to recent progress in controlling root‐parasitic *Striga* species, which are representative of root‐parasitic weeds in general.

## 2 PROGRESS IN *STRIGA* CONTROL

This review recognizes that there has been an outstanding output of basic research on root‐parasitic weeds that is very important and very cogent for understanding the parasitism from physiological, molecular and genetic perspectives. The information now being generated was lacking when the author became interested in parasitic weeds over three decades ago, and is still needed to develop novel control strategies. Conversely, as this review concludes, the present applicability of these results to the field is far less impressive, and improvements are suggested where called for.

### 2.1 Breeding solutions

‘It's like déjà vu all over again!’

(attributed to Yogi Berra)

Two types of breeding solutions continue to be reported, combining land‐races, inbreds etc. based on actual physiological mechanistic data with almost random recombinations vs. using genome‐wide association studies (GWAS). Breeding makes sense (to this reviewer) with crops such as sorghum, the other millets and cowpeas, which co‐evolved with *Striga* for millennia, yet much of the breeding effort has been with maize, the predominant grain crop in sub‐Saharan Africa. Maize evolved by domestication in the *Striga*‐free Western Hemisphere, and only recently (on an evolutionary timescale) came to Africa. One expects that maize has fewer genes encoding resistance than the indigenous millets. These pessimistic expectations seem to have been borne out, and as explained by a reviewer of this manuscript who is more knowledgeable than this author: “The evidence shows that resistance to *Striga* in maize is a quantitative trait. Although several studies reported high broad‐sense heritability (>70%), more detailed studies found that SNPs (single nucleotide polymorphisms) identified with GWAS only explained 3–14% of phenotypic diversity. (e.g. Stanley *et al*.^1^). This suggests that it is unlikely that modifying a few genes would confer robust resistance, which also would explain why several of the studies cited in this article have not provided adequate *Striga* control.” Decades of mainly maize breeding efforts for S*triga* resistance at international centers have not had a major impact on yield per hectare compared with the enhanced yields gained by breeding elsewhere over the same period. There are some maize varieties and hybrids that are less affected by *Striga*, but there is enough *Striga* parasitizing them to replenish the seed bank. Perhaps the gene(s) responsible for this modicum of maize resistance regulate the level of abscisic acid produced as a result of *Striga* infection, as these genes are found in maize. It has long been thought that abscisic acid is at least in part responsible for the poisoning/bewitching response to *Striga*,^2^ although there was no correlation between leaf abscisic acid and *Striga* tolerance among sorghum lines (which have other mechanisms of resistance).^3^ The time has come to more fully understand the physiology of bewitching, as preventing it would reduce the damage caused by *Striga*. Whether this correlation occurs in maize should be checked to ascertain whether suppressing induced abscisic acid synthesis confers some tolerance, as this could be useful as a marker in breeding as well as a target for gene editing and engineering in maize. There was no correlation between a reduced level of extracted germination stimulant produced by 27 maize lines and their level of resistance,^4^ suggesting that resistance is not due to differences in induced *Striga* germination.

Breeding based on the physiological characters of the breeding material was considerably more successful in other *Striga* host crops than maize once some lessons were learnt. As we will see later, these messages seem to have been forgotten by contemporary researchers performing gene editing. The group of Gebisa Ejeta screened sorghum for reduced production of the host root‐exuded *Striga* germination stimulant. Pot experiments showed excellent resistance under low *Striga* seeding, and the material provided adequate control in moderately infested fields, but the low stimulant lines were decimated where *Striga* field infestations were heavy and resistance was most needed. When stimulant secretion is reduced, it lowers the distance the stimulant can diffuse in the soil and still be effective. With high infestation levels, there are enough *Striga* seeds in the seedbank close enough to crop roots to be induced to germinate with reduced levels of stimulant. The Ejeta group continued breeding, combining the low stimulant lines with a line with reduced haustorium formation and a line where penetration was reduced. The resulting material was far more robust under high infestation levels.^5^ The problem with this approach is that one must deal with three or more recessive genes when attempting to transfer the resistance into locally adapted material. Only recently has one of the resistant mutant traits been mapped to a gene for resistance, a marker that detects all natural *Igs*1 mutations having low *Striga* germination stimulant in sorghum.^6^ If markers for the other genes responsible for resistance were to be similarly isolated, this could assist in breeding robust resistance in sorghum, as well as being used in molecular methods to deal with *Striga* in sorghum and other crops, as discussed in Sections 2.6. and 2.7.

### 2.2 Suicidal germination stimulants

*Striga* seeds are capable of remaining dormant in soil for a decade or more until a host root approaches. It has long been a goal to artificially induce the germination of parasitic weeds and have them suicidally die before planting a host crop. There has been more success with *Orobanche/Phelipanche* spp. than with *Striga* spp., achieved by planting unaffected ‘trap’ or ‘catch’ crops that produce germination stimulant to reduce the seedbank in rotation with an affected host crop.

Decades of research have gone into synthesizing stimulants that induce suicidal germination, and many have been highly effective in laboratory situations, but until recently, none have been successful in the field due to their very short half‐lives. This has changed by using an undisclosed proprietary formulation that keeps the germination stimulants active in soil for a sufficient duration to be effective. Experiments in western Kenya using the formulated stimulants led to *Striga*‐free maize plots within three heavily infested field sites.^7^ Is this practical? One must look carefully at the experimental protocol. The experiments began with the first rains needed to hydrate the *Striga* seed and continued over a 2‐week period (‘preconditioning’) before applying the newly formulated stimulants. After preconditioning the seed would typically either germinate when chemically induced by a compatible host root or redry until next preconditioned. After preconditioning there were two spaced applications of the formulated stimulants and the *Striga* was given time to germinate and die due to lack of host. The maize crop was finally planted, almost 3 months after the onset of rainfall, when maize is usually planted at the beginning of a rainy season. As yield comparisons were not presented, the yield penalty for such late planting is unclear, but was almost certainly significant.

The synthetic germination stimulants are all highly complex compounds (although much simpler than the earliest ones made) and researchers continue to synthesize new ones.^8^, ^9^ These complex molecules must be expensive to synthesize. It has puzzled this scientist that no efforts seem to have been made to formulate inexpensive ethylene to be released slowly – ethylene and its precursor are long known to induce *Striga* germination without a host.^10^

### 2.3 Push‐pull

This highly hyped strategy of intercropping the allelochemical‐secreting perennial legume *Desmodium* with maize that prevents *Striga* and repels insects (push) with a border of Napier or Sudan grass that attracts stem‐borers (pull) has not been widely adopted. It takes 2 years to establish the *Desmodium* stand, and intercropping is labour intensive and the fields are not amenable to the mechanised harvest of the crop or the push‐pull species. *Desmodium* provides legume fodder for animals, benefiting the nutrition of the subsistence farmers, but *Desmodium* will not bring them out of subsistence into highly productive agriculture, just to more labour‐intensive farming. A recent study to expand the the technology to Ethiopia showed that all the effort expended to establish the technology resulted only in an 80% reduction in emerged *Striga*,^11^ which reduces yield and replenishes the seedbank.

A considerable amount of basic research to elucidated the active allelochemicals that *Desmodium* produces: complex flavonoids such as uncinanone B and various di‐C‐glycosylflavones that stimulate suicidal germination and uncinanone C that inhibits *Striga* attachment.^12^ It would be difficult to chemically synthesize and use them economically. People might eventually try to find simplified versions of these allelochemicals that could be used as a mixture (possibly synergistic) of strigacides, or isolate the genes responsible for their synthesis and engineer them into crops with root‐specific promoters.

### 2.4 Herbicides

The technology of imazapyr herbicide coating of mutant acetolactate synthase herbicide‐resistant maize^13^ sucessfully controlled *Striga* and was commercially available until recently. The herbicide used was too expensive and was only available on coated seed. The herbicide often reduced crop stand and had negative carryover effects on following legume crops, and thus fell out of favour. There are much cheaper, less residual herbicides available that would work with the same mutant acetolactate synthase genetic material. These more appropriate herbicides could now be inexpensively sprayed post‐emergence on the whole field, controlling *Striga* as well as most other weeds, eliminating the need for traditional back‐breaking hand weeding. As labour costs are increasing in much of Africa, the labour saving could offset the herbicide cost.

A double‐encapsulated slow‐release formulation of the herbicide atrazine was constructed to control *Striga*,^14^ but no data were presented that it actually does so. As atrazine inhibits photosynthesis, the *Striga* control should only occur after emergence when the hemi‐parasite greens and becomes photosynthetic. This occurs shortly after the *Striga* has irreversibly bewitched most crops. This technology, if cost effective, might have utility in conjunction with other technologies by controlling emerging *Striga* that ‘escaped’, and thereby preventing seed bank replenishment. Indeed, a different nanocomposite slow‐release formulation of atrazine reduced emerged *Striga asiatica* counts, resulting in less damage to sugarcane.^15^ It is not clear when/where the herbicide was active, as no data were presented on attachments, the measurements of emergence were far apart, and emerged plants may have died and wilted after emergence and before counting.

### 2.5 Biocontrol agents

Various organisms have been isolated that impede *Striga* in one way or another. These include poorly thought out concepts such as using specially reared insects that can consume up to half of the seeds produced. Modeling suggests that there would be little reduction of the seedbank with such a low rate of seed destruction,^16^ and of course there would be no benefit to the crop during the cropping season used. The vast majority of the reports on biocontrol describe fungi or bacteria that are pathogenic to *Striga* and ‘show promise’. Such reports continue to appear.^17^

In this bleak background, the work of one group stands out, and their work resulted in a registered commercial product containing a *Striga*‐specific *forma specialis* of a *Fusarium oxysporum* that is available in more than one African country.^18^ They succeeded with an organism others had tested yet had been unsuccessful in developing the technology for field use. Success was achieved because this group fulfilled the four requirements for a successful biocontrol agent^19^: the fungus was mutated to become hypervirulent to increase its efficiency; they cultivated the inoculum inexpensively in liquid culture; they formulated the inoculum on the crop seed with sufficient nutrients for it until it encounters *Striga*; and they demonstrated to regulators that it is host specific and thus biosafe (despite regulators innate fear reaction on hearing ‘*Fusarium’*). This product is one of the only successful mycoherbicides currently being sold for control of any weed. There is a good possibility that a crop with this organism growing on its rhizoplane will also be protected from invasion by other root pathogenic fungi,^20^ possibly precluding the need for fungicide treatment of the crop seed.

### 2.6 Gene editing

Gene editing has been used by two groups to reduce the amount of strigolactone germination stimulant produced by the crop hosts of *Striga*. One group separately targeted three genes known to modulate strigolactone production, two with known functions and one mapping to reduced production, whose role is unknown.^21^ The resultant edited sorghum plants had undesirable increased tillering and at the single density of *Striga* infestation in the pots had significantly reduced numbers of emerging *Striga* plants,^21^ but this reduction of emerging *Striga* was from the low 30s per plant in the untreated controls to the low 20s per treated plant. This is not the level of resistance farmers expect or need. One wonders why the authors did not edit groups of genes to obtain cumulative or synergistic suppression, considering the senior author's previous studies showing the efficacy of stacking genes.^22^

A more targeted approach was used in an elegant series of experiments utilizing the latest tools of biochemistry and molecular biology to prevent the strigolactone germination stimulator from being secreted (instead of being synthesized as above) by host crop roots. This should overcome the increased tillering and other side effects of reducing total strigolactone biosynthesis in the previously described experiments. Two sorghum strigolactone transporters were identified by transcriptomic and functional analyses, and protein modeling was used to elucidate the conserved amino acids required for function of the transporters. Based on this information, single and double knockouts were generated by gene editing.^23^ In the 2‐year field experiment emerged *Striga* was *ca*. 70–80% suppressed in both the single knockouts and >95% in the double transporter knockout. The most important information for the growers comes from the field yield data: When infested with *Striga*, the unedited control plots incurred *ca*. 70% yield loss, while plots with the single and double mutants had yield losses of just 15% and 10%, respectively, compared to *Striga‐*free conditions.

How will this technology^23^ transfer from China where the research was performed to Africa where *Striga* resistance is needed? A comparison of conditions does not allow for rosy predictions, except possibly for the double knockout: the Chinese field experiments^23^ were performed with *Striga asiatica*, which is far less virulent than the predominant *Striga hermonthica* in Africa, and the seeding density used in the Chinese field experiments cited was 4200 seeds per square metre, orders of magnitude less than in heavily infested African fields. Thus there may be enough *Striga* seeds in African fields close enough to the gene‐edited host root to be stimulated to germinate by the minuscule amount of stimulant secreted.

As there normally is enhanced strigolactone secretion under phosphate deficiency, one wonders whether these gene‐edited plants will require more phosphate fertilizer than unedited plants when the plants are cultivated in the non‐ideal situation of the nutrient deficient farmers' fields, another unanswered question. The authors should remember the earlier studies where it was found that breeding low stimulant producing lines were only useful when genetically stacked with resistances at later stages.^5^ Equally elegant gene editing to what has been performed so far is needed to edit the prevention of haustorial formation and penetration, as well as establishment of the parasite. All the edited traits should then be stacked together to provide robust *Striga* control that would be recalcitrant to the parasite evolving resistance.

### 2.7 Transgenesis

There are no field data where this technology that entails engineering a gene encoding a needed trait along with its controlling elements has been used to deal with Striga . Unlike editing where pre‐existing traits are modified, with transgenesis the needed traits can be derived and modified from any organism that may bear the trait. Transgenic technologies also have the advantage over gene editing in ease of transferring the traits to locally adapted material. Transgenic traits are inherited dominantly whereas edited traits are inherited recessively. Additionally, if more than one trait is engineered into a single locus, the block of traits may be inherited dominantly as a single group without the individual traits segregating from each other. Such a technology has great potential to utilise accrued basic research on *Striga* and should not be ignored.

There is transgenic maize in commerce that surely would allow inexpensive *Striga* control: the 30% of the world's maize that is transgenically resistant to the herbicide glyphosate and is cultivated on 60 million hectares. There are no reports that it has been tested in *Striga*‐infested areas. It was long ago demonstrated that spraying this herbicide on transgenic‐resistant crops infested with the related *Philipanche aegyptiaca* controlled the parasite,^24^ and there is no reason to expect that this technology would not work with glyphosate‐resistant transgenic maize infested with *Striga*.

There is other recent basic research that might be used in *Striga* control: specific host‐secreted phenolic chemicals provide *Striga* with the signal to form haustoria, i.e. they are ‘haustorium‐inducing factors’.^25^ These lignin precursors are used to produce the lignins in the *Striga* root tips that provide the rigidity needed in forming haustoria.^26^ Inhibiting the secretion of these phenolic lignin precursors would presumably prevent formation of functional haustoria. This would require very precise construction of genetic engineering constructs that would allow the phenolic syntheses to continue in the crop roots but prevent their secretion. Localized lignification of the host root is an important aspect of host resistance by stopping the *Striga* penetration peg.^27^ Such results might be obtained performing research similar to the gene editing of transporters that limited strigolactone efflux described in Section 2.6, or by transgenically targeting the production of the specific transporter using RNAi or antisense systems with root‐specific promoters.

It has long been known that short sequences of double‐stranded RNA matching a pest‐specific gene when engineered into a host could translocate into and replicate in the pest after contact and suppress the pest gene. This line of research, termed host‐induced gene silencing (HIGS), initially began with nematodes and spread to insects and pathogens as well *Orobanche*. The HIGS technology went out of favour as the ‘promising’ results were statistically significant but agronomically insignificant because the levels of control of all the target pests was insufficient. This changed when constructs targeting three different genes were simultaneously used to suppress a mycotoxin along with the pathogen that produced it.^22^ It was suggested that the use of stacked multiple targets in a HIGS construct, along with better choice of target genes based on knowledge of temporal transcriptomics, might also provide agronomically significant parasitic weed control.^28^

That better HIGS target genes would provide better control was recently demonstrated by targeting various proteins secreted by the related *Phelipanche aegyptiaca* during attachment.^29^ Each one of the genes encoded was targeted separately and a few of them severely reduced the number of attachments as well as parasite size. These results might be sufficient for controlling *Orobanche*, which does not bewitch its host, unlike *Striga*. It is hoped that we will soon see a report where HIGS targeting the best three genes has been combined into a single HIGS construct, as this would surely provide even better control with the added advantage that it would be virtually impossible for the parasite to simultaneously mutate the three genes needed for it become resistant to the stacked HIGS RNAs. This HIGS technology could be transferred to *Striga* as similar ‘secretome’ proteins have also been identified that are secreted by *Striga* in its host during early penetration.^30^ The genes encoding such proteins may well be excellent targets for the HIGS technology.

## 3 CONCLUDING REMARKS

Typically, when photographs of excellent *Striga* control on crops are presented, they impressively show nearly *Striga*‐free patches due to the treatments *vs* beautifully flowering *Striga* in the untreated controls. One might erroneously get the impression that *Striga* is the only weed problem from these pictures. The fields were hand‐weeded of all other weeds prior to photographing. The other weed problems were dealt with by back‐breaking work, mostly performed by women and girls. Because a woman can only weed so much area in a day, much of the general weed control on subsistence farms is performed too late, when the other weeds have heavily reduced potential yield. It seems unethical to have women and girls hand weed when there are herbicides that can safely and inexpensively replace laborious and time‐consuming hand weeding. Post‐emergence herbicides are needed that will control both underground *Striga* along with the plethora of other weeds. As noted above, this could be done with glyphosate‐resistant transgenic maize or with the mutant maize resistant to acetolactate synthesis‐inhibiting herbicides already available.

It is a pervasive problem that agricultural scientists are taught to deal with one variable (problem) at a time, where the farmers have to simultaneously deal with many constraints. *Striga* and other weeds are not their only problems. Farmers must also contend with insects, pathogens and rodents, as well as an increasingly erratic climate. Subsistence farmers cannot afford the chemical inputs to deal with all the pest issues. The best solutions that will assist them to upgrade from a subsistence economy to a highly productive are ones that provide them with genetic solutions (‘in seedo’ technologies) or seed treatments (‘on seedo’ technologies) that deal with as many constraints as possible.

Not only must we learn from past and present mistakes, we need to learn one from another. An example of such a case is insights from the chemically induced suicide germination research with its excellent *Striga* control but vastly delayed planting that could be implemented in developing better mycoherbicide seed treatments that further reduce the *Striga* seedbank. The mycoherbicide group might consider adding ethylene emitting bacteria^31^ to the mycoherbicide treatment to induce suicidal germination or to consider engineering their *Fusarium* with the genes needed for ethylene biosynthesis, which have been well characterized.^32^

As the subsistence agricultural world is composed of many different agro‐ecosystems, all novel solutions must be introgressed into locally adapted material. The seed company breeders have attempted to introduce the few genetic traits available, but as shown with *Striga* they are insufficient. African plant protection specialists pinpointed many constraints that are intractable to breeding solutions decades ago that might be amenable to genetic engineering.^33^ Unfortunately for Africa (and India), the need for genes controlling the fall‐armyworm can be added to the list of breeding intractable traits. In a recent review it was suggested that the most efficient way to deal with these multiple problems is to genetically engineer groups of genes conferring many of the needed traits onto single chromosomal loci, such that they would then be dominantly inherited together without segregating,^34^ easing introducing complexes of into elite locally adapted germplasm.

Luckily, many *Striga*‐infested countries now seem to be amenable to genetic engineering solutions to their local problems in light of increased world food insecurity and commodity grain price fluctuations. Much grain that had been imported from the West is now going into biofuels, causing an increase in world prices. One of the first proclamations of the present Kenyan government was that it is open to any modern genetic technology that safely solves problems.^35^ Many of the needed genes have been isolated, have proven to be effective when used singly or stacked, and are off‐patent. There is now a cadre of African scientists well‐trained to perform transgenesis and gene editing, and the lowered costs of using these technologies has made them more accessible.^36^

We also have questions of how applicable are solutions from one crop to another, from one locale to another, as well as how long given solutions will remain usable due to *Striga* species evolving resistance. There is ample evidence that the two major *Striga* species have a great amount of diversity both for locations and for the host crops attacked.^37^, ^38^ Clearly crops with multiple stacked solutions will be more likely to overcome the diversity issues.

Déjà vu research will not solve *Striga* or other constraints limiting food production in developing countries. We must make sure that we quickly utilize the recent and future results from basic research on *Striga* (and other constraints) to as quickly as possible increase food security in the most efficient and safe way possible, using the best technologies at our disposal.

## Data Availability

The data that support the findings of this study are available in the refereences cited in the manuscript.
